# Lycobetaine acts as a selective topoisomerase IIβ poison and inhibits the growth of human tumour cells

**DOI:** 10.1054/bjoc.2001.2142

**Published:** 2001-11

**Authors:** H U Barthelmes, E Niederberger, T Roth, K Schulte, W C Tang, F Boege, H-H Fiebig, G Eisenbrand, D Marko

**Affiliations:** Department of Chemistry, Division of Food Chemistry and Environmental Toxicology, University of Kaiserslautern, Erwin-Schroedinger Str. 52, Kaiserslautern, 67663; Medizinische Poliklinik, University of Würzburg Medical School, Klinikstr. 6–8, Würzburg, 97070; Tumor Biology Center at the University of Freiburg, Breisacher Str. 117, Freiburg i. Br., 79106; Institute for Experimental Oncology, Oncotest GmbH, Am Flughafen 8–10, Freiburg i. Br., 79110, Germany

**Keywords:** lycobetaine, ungeremine, topoisomerase IIβ, cleavable complex, clonogenic assay, gastric carcinoma

## Abstract

The phenanthridine alkaloid lycobetaine is a minor constituent of Amaryllidaceae. Inhibition of cell growth was studied in the clonogenic assay on 21 human tumour xenografts (mean IC _50_ = 0.8 μM). The growth of human leukaemia cell lines was also potently inhibited (mean IC _50_ = 1.3 μM). Athymic nude mice, carrying s.c. implanted human gastric tumour xenograft GXF251, were treated i.p. with lycobetaine for 4 weeks, resulting in a marked tumour growth delay. Lycobetaine was found to act as a specific topoisomerase IIβ poison. In the presence of calf thymus DNA, pure recombinant human topoisomerase IIβ protein was selectively depleted from SDS-gels, whereas no depletion of topoisomerase IIα protein was observed. In A431 cells immunoband-depletion of topoisomerase IIβ was induced, suggesting stabilization of the covalent catalytic DNA-intermediate in living cells. It is reasonable to assume that this mechanism will cause or at least contribute significantly to the antitumour activity. © 2001 Cancer Research Campaign   http://www.bjcancer.com


*British Journal of Cancer* (2001) **85**(10), 1585–1591
© 2001 Cancer Research Campaign
doi: 10.1054/ bjoc.2001.2142, available online at http://www.idealibrary.com on IDEAL

### Lycobetaine acts as a selective topoisomerase IIβ poison and inhibits the growth of human tumour cells

**HU Barthelmes[2], E Niederberger[1], T Roth[4], K Schulte[1], WC Tang[1], F Boege[2], H-H Fiebig[3,4], G Eisenbrand[1] and D Marko[1]**

[1]Department of Chemistry, Division of Food Chemistry and Environmental Toxicology, University of Kaiserslautern, Erwin-Schroedinger Str. 52, 67663 Kaiserslautern; [2]Medizinische Poliklinik, University of Würzburg Medical School, Klinikstr. 6–8, 97070 Würzburg; [3]Tumor Biology Center at the University of Freiburg, Breisacher Str. 117, 79106 Freiburg i. Br.; [4]Institute for Experimental Oncology, Oncotest GmbH, Am Flughafen 8–10, 79110 Freiburg i. Br., Germany

**Summary** The phenanthridine alkaloid lycobetaine is a minor constituent of Amaryllidaceae. Inhibition of cell growth was studied in the clonogenic assay on 21 human tumour xenografts (mean $IC_{50}$ = 0.8 μM). The growth of human leukaemia cell lines was also potently inhibited (mean $IC_{50}$ = 1.3 μM). Athymic nude mice, carrying s.c. implanted human gastric tumour xenograft GXF251, were treated i.p. with lycobetaine for 4 weeks, resulting in a marked tumour growth delay. Lycobetaine was found to act as a specific topoisomerase IIβ poison. In the presence of calf thymus DNA, pure recombinant human topoisomerase IIβ protein was selectively depleted from SDS-gels, whereas no depletion of topoisomerase IIα protein was observed. In A431 cells immunoband-depletion of topoisomerase IIβ was induced, suggesting stabilization of the covalent catalytic DNA-intermediate in living cells. It is reasonable to assume that this mechanism will cause or at least contribute significantly to the antitumour activity. © 2001 Cancer Research Campaign http://www.bjcancer.com

**Keywords**: lycobetaine; ungeremine; topoisomerase IIβ; cleavable complex; clonogenic assay; gastric carcinoma

The phenanthridine alkaloid lycobetaine (ungeremine, Figure 1) has been isolated as a minor constituent from several plant species of the Amaryllidaceae family (Owen et al, 1976; Ghosal et al, 1986; Lee et al, 1994). Lycobetaine has been reported to exhibit growth inhibitory properties in vitro (Wang et al, 1987; Ghosal et al, 1988) and to show significant cytotoxic activity against Ehrlich ascites carcinoma, ascites hepatoma, the leukaemias L1210 and P388, Lewis lung carcinoma and Yoshida ascites sarcoma in mice or in rats after i.p. injection (Zhang et al, 1981). In nude mice with gastric cancer, lycobetaine has been reported to extend the survival time and to decrease the tumour size (Wu et al, 1988). However, the underlying mechanism of action has not been elucidated yet. The present study addresses potential mechanisms of action of lycobetaine.

### MATERIALS AND METHODS

### Materials

Lycobetaine was obtained by oxidation of lycorine with selenium dioxide (Ghosal et al, 1986; He and Weng, 1989). Lycorine was isolated from bulbs of *Sternbergia lutea* (Amaryllidaceae) (Evidente et al, 1984) and characterized by HPLC and $^1$H-NMR spectroscopy in comparison with reference material kindly provided by Dr B Xu, Shanghai Institute of Materia Medica, Chinese Academy of Sciences, China. All chemicals used were of research grade.

*Received 24 January 2001*
*Revised 20 July 2001*
*Accepted 24 August 2001*

*Correspondence to:* D Marko

### Cell lines

The large cell lung tumour xenograft LXFL 529L was established in serial passage onto nude mice, NMRI genetic background (Berger et al, 1992) from which a permanent cell line was developed. The cell lines LXFL 529L, HL60, U937, K562 and Molt4 were cultured at 37˚C (5% $CO_2$ and 98% humidity) in RMPI 1640 medium with addition of 1% penicillin/streptomycin and 10% fetal calf serum (FCS). Medium, FCS and penicillin/streptomycin were obtained from Gibco Life Technologies (Karlsruhe, Germany). Cells were subcultured twice weekly and were routinely tested for the absence of mycoplasm contamination.

### Sulforhodamine B assay

Growth inhibition was determined using the sulforhodamine B assay (Skehan et al, 1990) with slight modifications as described previously (Marko et al, 2001).

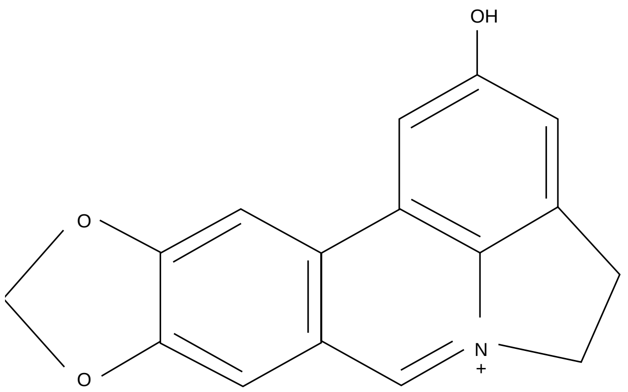

**Figure 1** Structure of lycobetaine

### Clonogenic assay

18 human tumours established in serial passage in nude mice (NMRI nu/nu strain) and 3 cell line-derived xenografts were used (Berger et al, 1992). The clonogenic assay was performed as a modified 2-layer soft agar assay as described previously (Drees et al, 1997). Inhibition of colony formation was expressed as treated/control × 100 (T/C%). $IC_{50}$ and $IC_{70}$ values were determined by plotting compound concentration versus cell viability. Mean $IC_{50}$ and $IC_{70}$ values were calculated as described previously (Roth et al, 1999).

### In vivo testing

Fragments of the gastric cancer GXF251 were implanted subcutaneously in both flanks of female nude mice (NMRI nu/nu strain). When tumours were approximately 3 × 3 mm, mice were randomly assigned to treatment and control groups. Lycobetaine was administered i.p. dissolved in 0.9% NaCl. Group 1 was treated twice weekly for 4 weeks with 60 mg $kg^{-1}$ bw. Body weight and tumour diameters were measured twice weekly. Tumour volumes were calculated from 2 perpendicular diameters measured by calipers ($a \times b^2/2$) and the optimal T/C as well as the tumour growth delay in days was determined (Drees et al, 1997). All tests were performed according to UKCCCR Guidelines for the Welfare of Animals in Experimental Neoplasia (Workman et al, 1998).

### Single cell gel electrophoresis (Comet assay)

HL60 cells ($1 \times 10^6$ $ml^{-1}$) were incubated for 3 h in RPMI 1640 medium containing 10% FCS in the presence or absence of the test compounds. Single cell gel electrophoresis (SCGE) was performed according to the method of Gedik (Gedik et al, 1992). DNA-strand breaks were detected by fluorescence microscopy and quantified by using the comet assay II system (Perceptive instruments, Suffolk, UK).

### Biochemical analysis of topoisomerase-directed drug-effects

DNA-relaxation, and -cleavage we measured with pUC18-plasmid as a DNA substrate, whereas DNA-decatenation activity was measured with *C. fasciculata* catenated kinetoplast-DNA (obtained from TopoGen Inc, Columbus, Ohio). Human topoisomerases I, IIα, and IIβ were expressed in *S. cerevisiae* and purified by various chromatographic steps, as described by Knudsen et al, (1996). Assay conditions and electrophoretic separation of the reaction products were precisely described by Boege (1996). For the study of topoisomerase-directed drug effects in cells, we used human A431 epidermoid cells. Cell culture and immunoblotting of cellular DNA-topoisomerases was done as described in Meyer et al (1997). Gels were documented by digital photography. X-ray films were digitalized with a flatbed scanner. Since statistical analysis could not be applied to the data, we present examples of results representative of at least 3 experiments with similar outcome performed on different days and with different sets of enzymes or cells.

### Flow cytometry

LXFL529L cells were incubated for 72 h with compounds on 10 cm Petri-dishes in RPMI 1640 medium containing 10% FCS. Flow cytometry was performed as described previously (Marko et al, 1998).

### RESULTS

### Growth inhibition in vitro

The activity profile in the clonogenic assay with human tumour xenograft cells shows that lycobetaine inhibits the growth of the 21 tumour xenografts tested within a range of 2 nM up to 27.5 μM, with a mean $IC_{70}$ value of 3.3 μM (mean $IC_{50}$ = 0.8 μM, Figure 2). Substantial activity ($IC_{70} \leq 1$ μM) was observed against the 2 gastric carcinomas tested, the large-cell lung carcinoma LXFL529, the small-cell lung carcinoma LXFS538, the colon carcinoma CXF280, the ovarian carcinoma OVXF1023 and the pancreatic carcinoma PAXF546. Marked growth inhibition ($IC_{70} \leq 3$ μM) was also observed for 2 bladder carcinomas (BXF1299 and BXF1301), one melanoma (MEXF989) and the renal carcinoma RXF944LX. The mammary carcinomas (MX1, MCF7X) and the lung adenocarcinomas (LXFA289, LXFA526) appear to be more resistant to lycobetaine. This also applies to the other colorectal, ovarian, pancreatic and renal tumours tested, as well as the second melanoma, resulting in an overall heterogeneous response pattern for these tumours. Human leukaemia cell lines were also growth inhibited in the low micromolar range (mean $IC_{50}$ = 1.3 μM, SRB assay), as shown in Table 1. Similar results were obtained for the xenograft derived permanent human large-cell lung carcinoma cell line LXFL529L, tested for comparison (Table 1).

### Antitumour efficacy in vivo

The gastric tumour xenograft GXF251 was chosen for in vivo testing. Athymic nude mice, carrying s.c. implanted tumour xenografts, were treated i.p. twice weekly for 4 weeks (Table 2). This group received a total dose of 480 mg $kg^{-1}$, resulting in a growth inhibition of about 50%. The schedule was well tolerated, as judged by only marginal effects on body weight gain, however, a late toxic death (day 21) was recorded (Table 2).

### Induction of DNA strand breaks

HL 60 cells were incubated for 3 h with lycobetaine. DNA strand breaks were determined using the comet assay (Gedik et al, 1992) (Figure 3). The extent of DNA damage induced correlates with the fluorescence intensity of the comets, quantified as tail intensity. Incubation with lycobetaine for 3 h resulted in 32% slightly and 10% severely damaged cells at 5 μM, whereas at 10 μM 82% slightly and 6% severely damaged cells were observed, with only 12% of the cells remaining intact.

**Table 1** Effect of lycobetaine on the growth of human tumour cell lines

| Cell line | Growth inhibition[a] $IC_{50}$ (μM) |
|---|---|
| HL60 | 1.3 ± 0.5 |
| Molt 4 | 0.7 ± 0.4 |
| K 562 | 0.8 ± 0.03 |
| U 937 | 2.5 ± 0.1 |
| LXFL 529L | 1.2 ± 0.1 |

[a]Growth inhibition of human tumour cell lines was determined using the sulforhodamine B assay, incubation time 72 h. $IC_{50}$ values were calculated as survival of treated cells over control cells × 100 (T/C%). Values are given as mean ± SD of 2–4 independent experiments, each done in quadruplicate.

*© 2001 Cancer Research Campaign*

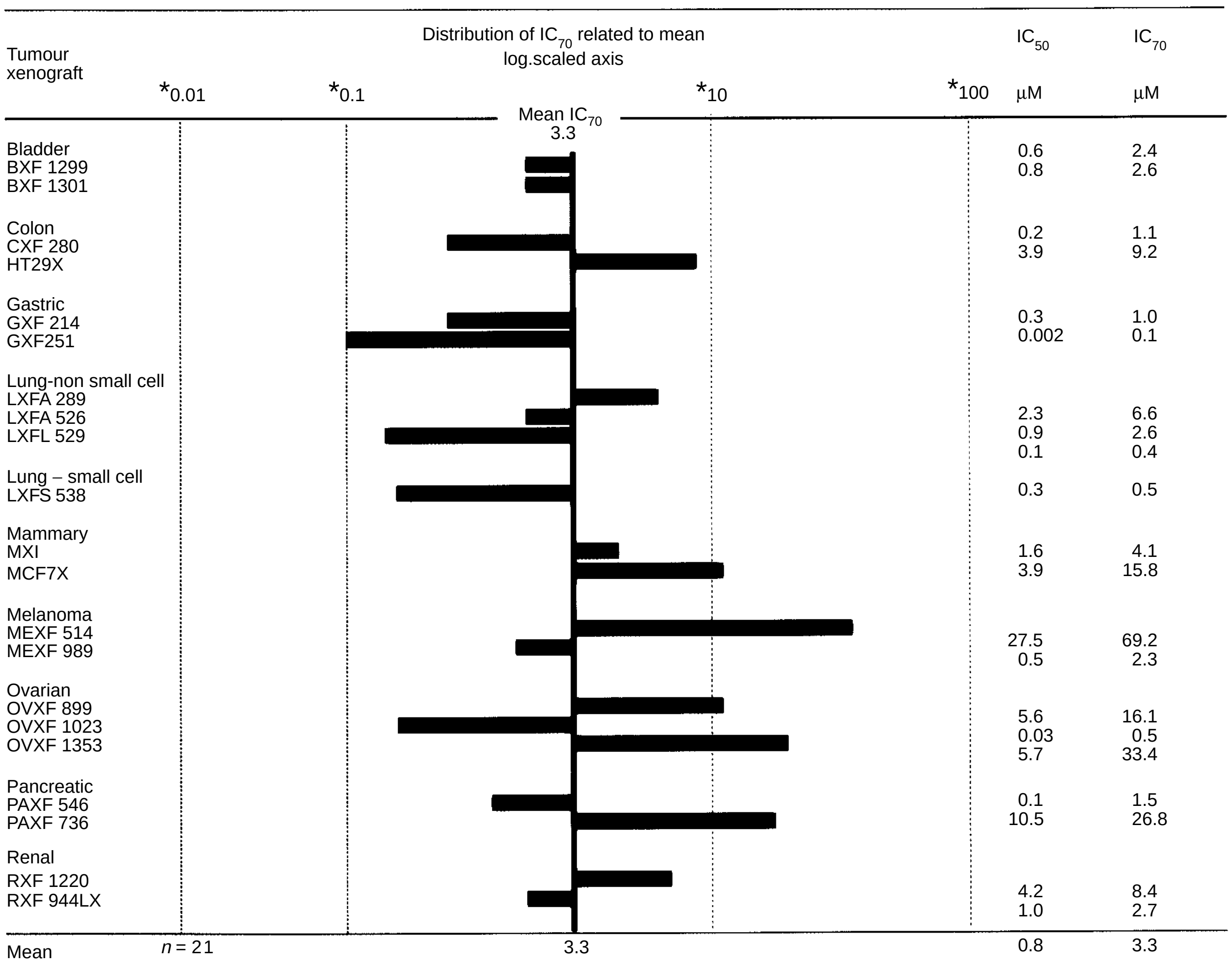

**Figure 2** Activity profile of lycobetaine on the growth of human tumour xenografts in the clonogenic assay. Cell suspensions of human tumour xenografts, grown onto nude mice, were seeded in soft agar and cultivated for 6–15 days, depending on the doubling time of the tumour stem cells. Lycobetaine was applied continuously in various concentrations. After the incubation time and staining with a vital dye, tumour colonies with a diameter > 50 µm were counted with an automatic image analysis system. Variations of individual $IC_{70}$s (drug concentration reducing colony formation to 30% of control values) from the mean value are shown as bars in the logarithmically scaled axis. Bars to the left demonstrate $IC_{70}$s lower than the mean value, bars to the right demonstrate higher values

**Table 2** Tumour growth inhibition in vivo[a]

| Group | Animals/tumours per group | Dose (mg kg$^{-1}$) | Schedule in days (i.p.) | Toxic deaths | Body weight change (%) on day | | | Optimal T/C | Tumour doubling time (days) | Tumour growth delay (days) |
|---|---|---|---|---|---|---|---|---|---|---|
| | | | | | 14 | 21 | 28 | | | |
| Control | 6/8 | 0 | 5 times/week for 4 weeks | 0 | + 8 | +5 | +1 | – | 7.2 | – |
| Group 1 | 5/10 | 60 | twice weekly for 4 weeks | 1 (day 21) | –1 | –4 | –11 | 50.6 | 9.4 | 2.1 |

[a]Treatment of nude mice bearing subcutaneously the human gastric tumour xenograft GXF251 started at a tumour size of approximately 80 mm$^3$ (day 20 after tumour implantation). Lycobetaine was dissolved in 0.9% NaCl and applicated by i.p. injection. Mice in the control group were treated with the vehicle (0.9% NaCl).

### Lycobetaine stimulates DNA-cleavage by topoisomerase IIβ but not -α

In view of the pronounced DNA damaging effect of lycobetaine in the comet assay, we suspected that the drug might act as a stabilizer of covalent DNA intermediates of topoisomerase I and/or II. To test this hypothesis, we incubated pUC18 plasmid DNA with pure recombinant human topoisomerases and various concentrations of lycobetaine. Controls included camptothecin or VM-26 as typical poisons of topoisomerase I or II, respectively. Reaction products were electrophoresed in the presence of ethidium bromide in order to resolve relaxed and supercoiled as well as linearized and nicked plasmid forms. Figure 4A shows a typical result obtained with topoisomerase I. Obviously the enzyme alone

© 2001 Cancer Research Campaign 

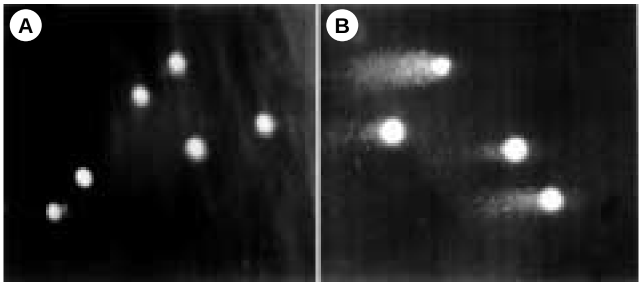

**Figure 3** Single-cell gel electrophoresis (comet assay). HL 60 cells were treated for 3 h with lycobetaine. Subsequent lysis and single cell gel electrophoresis were performed as described in Materials and methods. (**A**) Control. (**B**) Lycobetaine 10 μM, 3 h. The categories of DNA damage were separated according of the proportion of extranuclear fluorescence into 'not damaged' (proportion of the extranuclear fluorescence < 17%), slightly damaged (extranuclear fluorescence 17–60%) and severely damaged (> 60%)

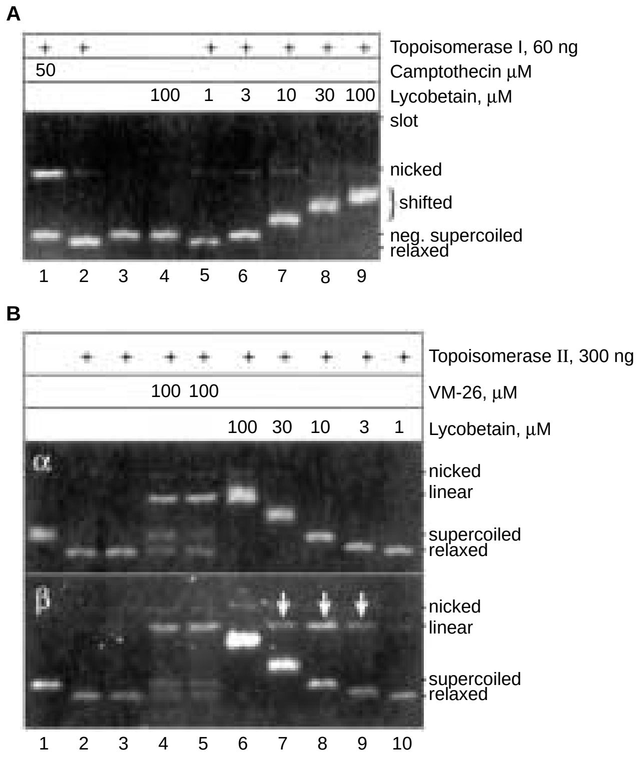

**Figure 4** Topoisomerase DNA-cleavage assays. 60 ng topoisomerase I (**A**) or 300 ng topoisomerase IIα (**B**, top) or 300 ng topoisomerase IIβ (**B**, bottom) were incubated with or without 200 ng pUC18 DNA at 37˚C. Camptothecin or lycobetaine were added to the incubations as indicated. Reactions were stopped after 30 min with 1% SDS. Samples were digested with proteinase K, and subjected to submarine 1% agarose gelelectrophoresis in the presence of ethidiumbromide. UV- transilluminated gels were documented by digital photography. This is a representative result of 5 identical experiments with similar outcome. Arrows in the bottom panel of (**B**) indicate linearization of plasmid DNA promoted by a concerted action of topoisomerase IIβ and lycobetaine

(lane 2) relaxed the plasmid completely, whereas 50 μM camptothecin (lane 1) inhibited the relaxation reaction and at the same time induced nicking of a considerable portion of the substrate. These observations are in a perfect agreement with the known fact that camptothecin promotes topoisomerase I-mediated DNA single-stranded cleavage. When various concentrations of lycobetaine ranging from 1–100 μM (lanes 5–9) were tested in a similar fashion, it became apparent that lycobetaine did not promote plasmid nicking in the same way as did camptothecin. Thus, it can be concluded that lycobetaine does not damage DNA via stimulating topoisomerase I-mediated DNA cleavage. It should be noted that lycobetaine shifted the closed circular plasmid in a dose-dependent fashion. The shift was clearly also dependent on topoisomerase I. It was not seen with lycobetaine and DNA alone (lane 4), but also appeared in coincubations of topoisomerase II and lycobetaine (Figure 4B, lanes 6–9). Since the reaction products were thoroughly digested with proteinase before electrophoresis, the apparent band shifts cannot be due to the binding of topoisomerases to the DNA. They could, however, reflect the introduction of positive supercoils into the plasmid by topoisomerase I or II, which might be driven by the DNA-intercalating properties of lycobetaine, and, thus, be dependent on lycobetaine and topoisomerase. As that may be, the phenomenon was not further pursued, because it seemed irrelevant with respect to the molecular basis of the DNA-damaging properties of lycobetaine.

Figure 4B shows typical results of DNA-cleavage experiments with topoisomerase IIα (top) or -β (bottom). Most notably, lycobetaine clearly stimulated topoisomerase II-mediated DNA double- stranded cleavage (indicated by plasmid-linearization). However, it did so only in reactions containing the β-isoform and not in reactions containing the α-isoenzyme (lanes 7–9, compare top to bottom). The dose–response relationship of this effect was bell shaped (maximum at 10–30 μM), as to be expected from a substance that has also DNA-intercalating properties. The apparent selectivity of lycobetaine for the β-isoform of topoisomerase II was clearly opposed by the non-selective effect of the classic topoisomerase II poison VM-26, which stimulated DNA-cleavage of both isoenzymes (lanes 4 and 5). Taken together, these results suggested that lycobetaine might be a β-selective topoisomerase II-poison.

### Lycobetaine stabilizes the DNA-linkage of topoisomerase IIβ but not -α

To corroborate these findings, we approached the problem from the protein side, studying depletion of topoisomerase II proteins from SDS gels by drug-induced DNA-linkage. For this purpose we incubated pure recombinant human topoisomerase IIα or -β with calf thymus DNA in the presence or absence of drugs, stopped the reaction with SDS, and subjected these samples to SDS-polyacrylamide gel electrophoresis followed by Coomassie staining. Figure 5, lane 1 shows that 2 μg topoisomerase IIα (top) or -β (bottom) alone were readily detectable by the protein staining. The protein bands of both isoenzymes were only marginally diminished upon incubation with 6 μg calf thymus DNA (Figure 5, lane 2), whereas coincubation with DNA and VP-16 (lane 3) or VM-26 (lane 4), which are both established topoisomerase II poisons, effected a more or less complete removal of the protein bands of both isoenzymes from the gel, because the covalent enzyme–DNA complexes stabilized by these drugs are too large to enter the gel. A similar extent of band-depletion was also obtained with lycobetaine. However, in this case only topoisomerase IIβ became depleted whereas the α-isoform was not affected (Figure 5, lanes 6 and 7, compare top to bottom). Again, the lycobetaine effect on the DNA linkage of topoisomerase IIβ had a maximum between 10–30 μM and decreased at higher concentrations (Figure 5, lane 8).

 *© 2001 Cancer Research Campaign*

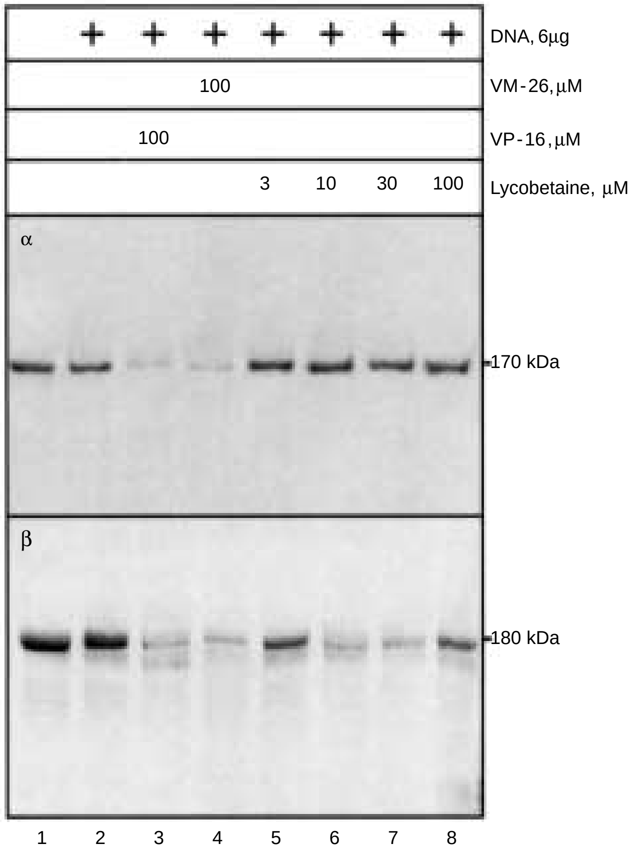

**Figure 5** Protein band depletion of pure topoisomerase II. 2 µg of pure recombinant human topoisomerase IIα (top) or -β (bottom) were mixed with 6 µg calf thymus DNA. Lycobetaine, VM-26, or VP-16 were added, as indicated. The sample in lane 1 was without DNA. Samples were incubated at 37°C for 30 min. Incubations were stopped with 1% SDS. Samples were then subjected to SDS-gel electrophoresis on 5.5% polyacrylamide gels. Gels were stained with coomassie blue, transilluminated and documented by digital photography. This is a representative result of 3 identical experiments with similar outcome

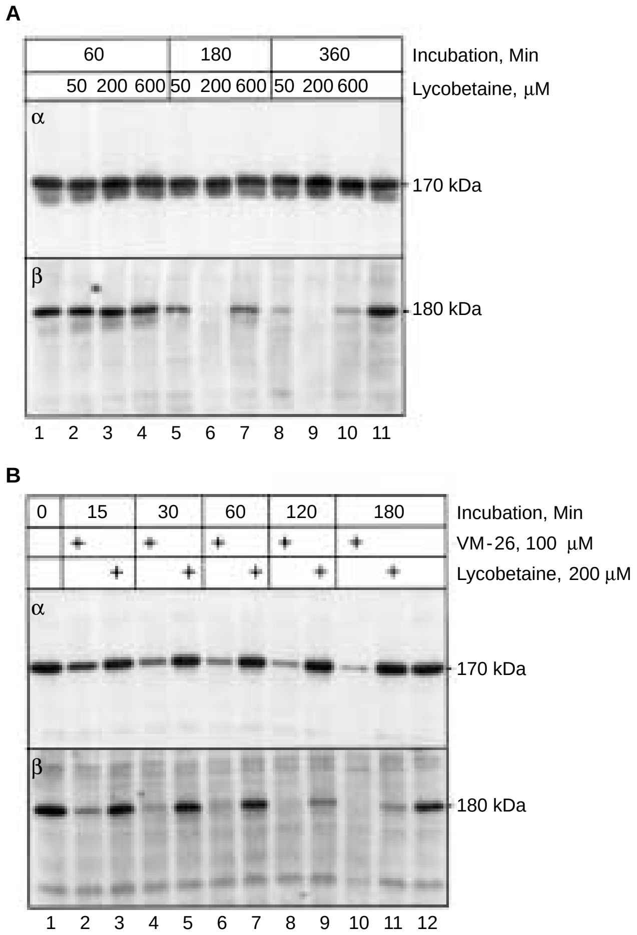

**Figure 6** Immunoband depletion of topoisomerase IIα and -β in A431-cells. Lycobetaine or VM-26 was added at the concentrations indicated to the medium of non-confluent monolayer cultures of $10^5$ A431 cells and culture was continued for the indicated periods of time. Subsequently the cells were lysed with 1% hot SDS and the cell lysates were rapidly applied to SDS-gel electrophoresis on 5.5% polyacrylamide gels, followed by Western blotting and immunostaining with rabbit peptide antibodies specific for the carboxyterminus of human topoisomerase IIα (top panels) or -β (bottom panels). Controls (lanes 1 and 11 in (**A**) and lanes 1 and 12 in (**B**)) were treated similarly without addition of drugs

### Lycobetaine promotes selective immunoband-depletion of topoisomerase IIβ in cells

In summary, the data shown in Figures 4 and 5 strongly suggest that lycobetaine might be a topoisomerase II poison selective for the β-form of the enzyme. As a next step we investigated whether it would also target topoisomerase IIβ in living cells. For this purpose, we added lycobetaine to the cell culture medium of A431 cells and assessed subsequently by immunoblotting the cellular pools of free topoisomerase IIα and -β that were not linked to DNA as a consequence of drug exposure. Drug-induced DNA-linkage of the cellular topoisomerase II can be determined as a loss of the respective protein band, because the DNA-linked enzymes are retained in the application slot together with the genomic DNA. In the absence of drug (Figure 6A, lanes 1 and 11) topoisomerase IIα (top) and -β (bottom) were readily detectable in the cells during the entire time-frame of the experiment. Upon exposure to lycobetaine topoisomerase IIα was not at all affected, whereas the signal of topoisomerase IIβ became clearly diminished (Figure 6A, lanes 6 and 9, compare bottom and top). The disappearance of the topoisomerase IIβ could in principle be due to proteolysis of the enzyme. However this is unlikely, because typical proteolytic degradation products are not apparent on the blot. Considering in addition the effect of the drug on purified topoisomerase IIβ (Figures 4 and 5) it seems most likely that the disappearance of the topoisomerase IIβ bands shown in Figure 6 is due to the induction of DNA-linkage. Maximal depletions were obtained with 200 µM lycobetaine, whereas minor effects were seen at higher concentrations (Figure 6A, bottom, compare lanes 6 and 7 or lanes 9 and 10). Most notably, the onset of these effects seemed to be delayed by at least one hour (Figure 6A, bottom, lanes 2–4) and increased gradually during incubations between 3 to 6 hours. Moreover, the effective concentration range seemed to increase during prolonged incubation periods (Figure 6A, bottom, compare lanes 5 with 8 and 7 with 10). Figure 6B compares the kinetics of topoisomerase II band depletion by lycobetaine with those of the classical poison VM-26. It is quite obvious, that the 2 drugs act on a different time-scale. The onset of VM-26 is more or less instantaneous and maximal band depletion is already obtained after 30 min (Figure 6B, lane 4), whereas lycobetaine needs as long as 120 minutes to produce a similar effect (Figure 6B, lane 9). Moreover, lycobetaine acts selectively on topoisomerase IIβ, whereas VM-26 depletes topoisomerase IIα and β (Figure 6B, compare top and bottom).

*© 2001 Cancer Research Campaign* 

### Cell cycle distribution

A further prediction of this type of mechanism of action, would be a block in the $G_2$/M-phase of the cell cycle. To test that assumption, LXFL529L cells were incubated for 72 h with lycobetaine. In untreated cells, 11% of the cells were found in the $G_2$/M phase. In the presence of 5 μM lycobetaine, a slight increase of cells in the $G_1/G_0$ phase became apparent, concomitant with a significant loss of cells in S phase and a distinct increase of the $G_2$/M cells (25%). The arrest in $G_2$/M was strongly enhanced upon incubation with 7.5 μM lycobetaine (32%) and an increase of cells with reduced DNA staining compared to cells in $G_1$ was observed. In agreement with flow cytometry results, at 10 μM lycobetaine, cells showed a distinct shrinkage of the cell volume and condensation of the chromatin, features characteristic for apoptosis induction (data not shown).

### DISCUSSION

Our results show that lycobetaine is a potent inhibitor of human tumour cell growth in the colony forming and in the SRB assay. In the clonogenic assay, enhanced activity against gastric carcinomas was observed. Lycobetaine was also active on certain lung and ovarian xenografts (Figure 2). This is in line with reports about substantial activity of lycobetaine in clinical studies in the treatment of ovarian and gastric carcinomas, at a dosage that was not reported to lead to significant systemic toxicity (He and Weng, 1989). In mice bearing the gastric carcinoma GXF251, lycobetaine exhibits substantial antitumour activity with minor toxicity on a twice weekly schedule, implying tolerable weight loss and 1 late toxic death (day 21). The antitumour efficacy was enhanced when lycobetaine was applied daily (30 mg $kg^{-1}$), resulting in a higher total dose. This resulted in increased toxicity, as judged from body weight loss, however, no toxic deaths were observed (data not shown). For exploration of an optimal dosing schedule further experiments appear warranted.

Using fluorescence microscopy, we observed exclusive localization of lycobetaine in the nucleus of human LXFL529L cells, comparable to ethidium bromide and Hoechst H33258 (data not shown). Lycobetaine has been described previously already as a DNA intercalating agent with preference for G–C pairs (Wu et al, 1987; Liu et al, 1989; Gan et al, 1992; Chen et al, 1997). This is consistent with our finding on its efficient competition for ethidium bromide intercalation into calf thymus DNA (data not shown). Furthermore, lycobetaine was found to compete effectively with the minor groove-binding ligand Hoechst H33258 (data not shown). In the comet assay, intense DNA damage was observed after treatment of HL60 cells with lycobetaine for 3 h (Figure 3). Mechanistically, DNA damage might occur as a consequence of the intercalative/minor groove-binding properties of lycobetaine, or, alternatively, lycobetaine might target as well topoisomerases directly. Minor groove-binding ligands have been reported to be potent topoisomerase I inhibitors with a mechanism similar to camptothecin, which is trapping the DNA-topoisomerase I cleavable complex (Chen et al, 1993). A number of DNA intercalators such as adriamycin, amsacrine or etoposide have already been shown to represent potent topoisomerase II inhibitors (Davies et al, 1988). We present here several lines of evidence characterizing lycobetaine as a topoisomerase IIβ-selective poison. Firstly, it stimulates DNA-cleavage by topoisomerase IIβ but not by topoisomerase IIα (Figure 4). Secondly, in the presence of calf thymus DNA it depletes pure recombinant human topoisomerase IIβ protein from SDS-gels, whereas it does not deplete topoisomerase IIα protein under similar conditions (Figure 5). Thirdly, it induces topoisomerase IIβ-selective immunoband-depletion in A431-cells (Figure 6). The latter result suggests that the drug does indeed stabilize the covalent catalytic DNA-intermediate in living cells, although we cannot altogether exclude that it induces proteolysis of the enzyme. However, in the view of the data obtained with pure topoisomerase IIβ (Figures 4 and 5) this is a less likely alternative. It is reasonable to assume therefore that poisoning of topoisomerase IIβ will cause or at least contribute significantly to the DNA-damaging properties of lycobetaine that might be relevant for the antitumour activity of the drug. As to be expected from a topoisomerase II inhibitor, lycobetaine was found to induce in a concentration-dependent fashion arrest of LXFL529L lung carcinoma cells in the $G_2$/M-phase of the cell cycle with indication for the onset of apoptosis.

Recently, an analogue of the herbicide Assure has been described (XK 469, NSC 697887), representing a synthetic quinoxaline phenoxypropionic acid derivative (Gao et al, 1999), which was found to inhibit topoisomerase IIβ at mM concentrations. On the basis of our data, lycobetaine is at least 10-fold more potent than XK 469. Lycobetaine is rapidly taken up into cells and locates to the nucleus. However, in view of the delay observed for the full onset of topoisomerase IIβ inhibition, it cannot be excluded at present that intracellular transport or metabolism plays also a role.

### ACKNOWLEDGEMENTS

We thank A Genzlinger and H Zankl for their help with the flow cytometry measurements. The study was supported by the grant 0310938 of the Bundesministerium fur Bildung, Wissenschaft, Forschung und Technologie, Germany (to GE) and the grants Bo 910/3-1 and Bo 910/4-1 of the Deutsche Forschungsgemeinschaft (to FB).

### REFERENCES

Berger DP, Winterhalter BR and Fiebig HH (1992) Establishment and characterization of human tumor xenografts in thymus aplastic nude mice. *Contr Oncol* **42**: 23–46

Boege F (1996) Analysis of eukaryotic DNA topoisomerases and topoisomerase-directed drug effects. *Eur J Clin Chem Clin Biochem* **34**: 873–888

Chen AY, Chiang Y, Gatto B and Liu LF (1993) DNA minor groove binding ligands: a different class of mammalian DNA topoisomerase I inhibitors. *Proc Natl Acad Sci USA* **90**: 131–135

Chen JZ, Chen KX, Jiang HL, Lin MW and Ji RY (1997) Theoretical investigation on interaction binding of analogs of AT-1840 to double-stranded polynucleotide. *Proc Natl Sci* **7**: 329–335

Davies SM, Robson CN, Davies SL and Hickson ID (1988) Nuclear topoisomerase II levels correlate with the sensitivity of mammalian cells to intercalating agents and epipodophyllotoxins. *J Biol Chem* **263**(33): 17724–17729

Drees M, Dengler WA, Roth T, Labonte H, Mayo J, Malspeis L, Grever M, Sausville EA and Fiebig HH (1997) Selective antitumor activity *in vitro* and activity *in vivo* for prostate carcinoma cells. *Clin Cancer Res* **3**: 273–279

Evidente A, Iasiello I and Randazzo G (1984) An improved method for the large-scale preparation of lycorine. *Chem & Ind*, 348–349

Gan L, Liu XJ, Chen KL and Ji YY (1992) Computer simulation on the interaction between antitumor agent lycobetaine and DNA. *Gaojishu Tongxun* **2**: 30–33

Gao H, Huang KC, Yamasaki EF, Chan KK, Chohan L and Snapka RM (1999) XK469, a selective topoisomerase IIβ poison. *Proc Natl Acad Sci USA* **96**: 12168–12173

Gedik CM, Ewen SWB and Collins AR (1992) Single-cell gel electrophoresis applied to the analysis of UV-C damage and its repair in human cells. *Int J Radiat Biol* **62**: 313–320

*© 2001 Cancer Research Campaign*

Ghosal S, Kumar Y, Singh SK and Kumar A (1986) Chemical constituents of Amaryllidaceae. Part 21. Ungeremine and criasbetaine, two antitumor alkaloids from *Crinum asiaticum*. *J Chem Res Synop* 112–113

Ghosal S, Singh SK, Kumar Y, Unnikrishnan S, and Chattopadhyay S (1988) Chemical constituents of Amaryllidaceae. Part 26. The role of ungeremine in the growth-inhibiting and cytotoxic effects of lycorine: evidence and speculation. *Planta Med* **54**: 114–116

He HM and Weng ZY (1989) Structure-activity-relationship study of the new anticancer drug lycobetaine (AT-1840). *Acta Pharm Sinica* **24**: 302–304

Knudsen BR, Straub T and Boege F (1996) Separation and functional analysis of eukaryotic DNA topoisomerases by chromatography and electrophoresis. *J Chromatogr B Biomed Appl* **684**: 307–321

Lee KH, Sun L and Wang HK (1994) Antitumor agents. 147. Antineoplastic alkaloids from Chinese medicinal plants and their analogs. *J Chin Chem Soc* **41**: 371–384

Liu J, Yang SL and Xu B (1989) Characteristics of the interaction of lycobetaine with DNA. *Acta Pharmacol Sinica* **10**: 437–442

Marko D, Romanakis K, Zankl H, Fürstenberger G, Steinbauer B and Eisenbrand G (1998) Induction of apoptosis by an inhibitor of cAMP-specific PDE in malignant murine carcinoma cells overexpressing PDE activity in comparison to their nonmalignant counterparts. *Cell Biochem Biophys* **28**: 75–101

Marko D, Schätzle S, Friedel A, Genzlinger A, Zankl H, Meijer L and Eisenbrand G (2001) Inhibition of cyclin-dependent kinase 1 (CDK1) by indirubin derivatives in human tumour cells. *Br J Cancer* **84**(2): 283–289

Meyer KN, Kjeldsen E, Straub T, Knudsen BR, Hickson ID, Kikuchi A, Kreipe H and Boege F (1997) Cell cycle-coupled relocation of types I and II topoisomerases and modulation of catalytic enzyme activities. *J Cell Biol* **136**: 775–788

Owen TY, Wang SY, Chang SY, Lu FL, Yang CL and Hsu B (1976) A new antitumor substance lycobetaine (AT-1840). *Ko Hsueh Tung Pao* **21**: 285–287

Roth T, Burger AM, Dengler W, Willmann H and Fiebig HH (1999) Human tumor cell lines demonstrating the characteristics of patient tumors as useful models for anticancer drug screening. In: Fiebig HH, Burger AM (eds) *Relevance of tumor models for anticancer drug development*, Contrib Oncol Vol 42. pp 145–156 Basel: Karger

Skehan P, Storeng R, Scudiero D, Monks A, McMahon J, Vistica D, Warren JT, Bokesch H, Kenney S and Boyd MR (1990) New colorimetric cytotoxicity assay for anticancer-drug screening. *J Natl Cancer Inst* **82**: 1110–1112

Wang XW, Yu WJ, Shen ZM, Yang JL and Xu B (1987) Cytotoxicity of hydroxycamptothecin and four other antineoplastic agents on KB cells. *Acta Pharmacol Sinica* **8**: 86–90

Workman P, Twentyman P, Balkwill F, Balmain A, Chaplin d, Double J, Embleton J, Newell D, Raymond R, Stables J, Stephens T and Wallace J (1998) United Kingdom Co-ordinating Committee on Cancer Research (UK CCCR) Guidelines for the Welfare of Animals in Experimental Neoplasia (Second Edition). *Br J Cancer* **77**: 1–10

Wu H, Shen CY and Xu B (1987) Effect of lycobetaine on DNA circular dichroism. *Chin J Pharmacol Toxicol* **1**: 272–276

Wu YL, Wu YX, Yu CS, Zhang SY, Su ZC and Jiang SJ (1988) The cytocidal effect of AT-1840 and parvovirus H-1 on gastric cancer cells. *Shanghai Med J* **11**: 683–688

Zhang SY, Lu FL, Yang JL, Wang LJ and Xu B (1981) Effect on animal tumors and toxicity of lycobetaine acetate. *Acta Pharmacol Sinica* **2**: 41–45

*© 2001 Cancer Research Campaign*
